# Screening Ticks for Crimean–Congo Hemorrhagic Fever Virus and Aigai Virus in Greece

**DOI:** 10.3390/v18050483

**Published:** 2026-04-22

**Authors:** Katerina Tsioka, Smaragda Sotiraki, Danai Pervanidou, Styliani Pappa, Konstantina Stoikou, Annita Vakali, Chrisovaladou-Niki Kefaloudi, Christina Sapanidou, Panagiota Ligda, Angeliki Liakata, Anastasios Saratsis, Dimitrios Chatzidimitriou, Anna Papa

**Affiliations:** 1 National Reference Center for Arboviruses and Haemorrhagic Fever Viruses, Department of Microbiology, Medical School, Faculty of Health Sciences, Aristotle University of Thessaloniki, 54124 Thessaloniki, Greece; aik.tsioka@gmail.com (K.T.); s_pappa@hotmail.com (S.P.); klstoikou@hotmail.com (K.S.); christinasapanidou@gmail.com (C.S.); dihi@auth.gr (D.C.); 2 Veterinary Research Institute, Hellenic Agricultural Organization (ELGO)-DIMITRA, Campus ELGO, Thermi, 57001 Thessaloniki, Greece; sotiraki@elgo.gr (S.S.); giota.lig@hotmail.com (P.L.); angelaliak1997@gmail.com (A.L.); saratsis@elgo.gr (A.S.); 3 National Public Health Organisation (EODY), 15123 Athens, Greece; d.pervanidou@eody.gov.gr (D.P.); a.vakali@eody.gov.gr (A.V.); c.kefaloudi@eody.gov.gr (C.-N.K.)

**Keywords:** Crimean–Congo hemorrhagic fever virus, Aigai virus, tick, *Rhipicephalus bursa*, *Hyalomma* spp., surveillance, Greece

## Abstract

Ixodid ticks are vectors for a plethora of pathogens, including the Crimean–Congo hemorrhagic fever virus (CCHFV), which causes severe disease in humans. Two autochthonous CCHF human cases were reported in 2025 in Greece. The aim of the present study was to gain a better insight into the geographic distribution and prevalence of CCHFV and the related Aigai virus (AIGV) in ticks in Greece. Therefore, 680 ticks (135 *Hyalomma* and 545 *Rhipicephalus* ticks) collected during 2024 from livestock (sheep, goats, cattle) and from the environment were tested for CCHFV and AIGV. AIGV was detected in 12 adult *Rhipicephalus bursa* ticks (12/511, 2.3%), while all *Hyalomma* ticks and *R. bursa* nymphs were negative for both viruses. AIGV-positive ticks were collected in May and June from goats and sheep in two distantly located regional units of Greece. AIGV sequences from partial S RNA segment differ from the prototype AIGV strain (AP-92) by 10.3% and 1.4% at the nucleotide and amino acid level, respectively. Integrated surveillance studies are needed in ticks, humans, wild and domestic animals within a One Health framework to gain a better insight into the epidemiology of CCHF in Greece, while clinical research is needed to elucidate the impact of AIGV in public health.

## 1. Introduction

Crimean–Congo hemorrhagic fever virus (CCHFV) and Aigai virus (AIGV) are tick-borne viruses belonging to the genus *Orthonairovirus*. According to the latest report from the International Committee on Taxonomy of Viruses (ICTV), the genus *Orthonairovirus* is one of the eight genera within the family *Nairoviridae* (order *Hareavirales*, class *Bunyaviricetes*). It includes 52 species of tick-borne viruses with a tri-segmented, negative-sense, single-stranded RNA genome [1,2]. The medically most important orthonairovirus is CCHFV (species *Orthonairovirus haemorrhagiae*), which is transmitted mainly by ticks of the *Hyalomma* genus and causes a severe disease in humans (CCHF), with fatality rates reaching up to 30% [3,4]. AIGV was recently reclassified as a distinct species, named *Orthonairovirus parahaemorrhagiae*. The new species includes the Greek strain AP-92 and all related “AP-92 like” viruses, which until recently were assigned to CCHFV genotype Europe 2 (or genotype VI) [5,6]. AP-92 was initially isolated in 1975 from *Rhipicephalus bursa* ticks in Vergina village (ancient Aigai) in northern Greece [7]. CCHFV and AIGV circulate in nature in enzootic sylvatic cycles involving ticks and non-human vertebrate hosts, influenced by local ecological and husbandry practices.

The first autochthonous human CCHF case in Greece was reported in 2008 in the northeastern part of the country; the case had a fatal outcome [8,9]. Two additional CCHF cases were reported in 2025 in Thessaly, Greece (a fatal tick-borne index case and a secondary case involving a healthcare worker) [10]. In all three cases, the CCHFV sequences clustered within CCHFV genotype Europe 1 (genotype V) [8,10].

Several seroprevalence studies indicate that the overall CCHFV IgG positivity in the Greek human population is approximately 4% (range 0–14.2%), with significant differences between the eastern (mean 2.3%) and western (mean 9.2%) part of the country [11,12,13,14]. Numerous factors significantly affect CCHFV seroprevalence in humans, including age, sex, tick bites, agropastoral activities, altitude, and environmental drivers, such as land cover type, the density of transitional woodland/shrub land per person, and the ratio of livestock—specifically, goats, sheep and cattle—per person [11,15]. The high CCHFV seroprevalence in Greece, combined with the rarity of clinical cases (only two tick-borne cases in a 17-year period), suggests that most, if not all, seropositive individuals had contact with a non- or low-pathogenic orthonairovirus. Consequently, all studies conclude that tick studies are needed to identify the circulating orthonairovirus strains and unravel the mystery of CCHF epidemiology in Greece [11].

Previous studies conducted between 2012 and 2014 on ticks collected from livestock in Greece resulted in the detection of CCHFV genotypes Europe 1 and Europe 2 (reclassified as AIGV). Most Europe 1 sequences were obtained from *Rhipicephalus sanguineus* sensu lato, whereas most AIGV sequences were recovered from *R. bursa*. The number of *H. marginatum* ticks (the primary CCHFV vector) was low (0.5% of the collected ticks) and all tested negative [16,17]. A subsequent study reported the isolation of AIGV (Pentalofos strain) from a female *R. bursa* tick removed from a goat in northern Greece [18].

In the present study, we screened *Hyalomma* and *Rhipicephalus* ticks for CCHFV and AIGV to gain better insight into the geographic distribution and prevalence of these two orthonairoviruses in Greece.

## 2. Materials and Methods

The ticks were collected within the framework of the EU4Health OH SURVector project which aims to establish a monitoring system for tick-borne viral pathogens. We conducted a tick surveillance study to map the distribution of CCHFV and AIGV vectors and determine the prevalence of these viruses in the collected ticks. The survey covered all mainland NUTS2 regions (except Attica) and Crete; 20 farms per region were selected for seasonal sampling using a stratified random approach with equal allocation (sample size) per region. The sampling protocol was standardized across regions and host species, with five animals sampled per farm, a maximum of five ticks per animal, and additional flagging in the surrounding areas. Farms within each NUTS2 region were selected through convenience sampling, based on accessibility and willingness of farmers to participate. All *Hyalomma* spp. and *R. bursa* ticks were included in the study. Due to the fact that cattle in Greece are primarily farmed, goats and sheep were chosen for tick sampling. Regions with prior human cases or known seropositivity were not intentionally oversampled.

In 2024, a total of 680 *Hyalomma* spp. and *R. bursa* ticks were collected from livestock and the surrounding environment across 122 farms, spanning nine of the 13 administrative Regions of Greece. Sampling was conducted from March to December to encompass the peak seasonal activity of adult *H. marginatum* and *R. bursa* (May to September). These species were collected in 22 of the 31 screened regional units (RUs). The ticks were collected from 216 sheep, 167 goats, and 3 cattle, while 12 were collected through environmental flagging.

Ticks were identified to the developmental stage, sex, genus and species level under a stereomicroscope. Identification was based on morphological characteristics (e.g., scutum, capitulum, festoons, spiracular plates, and leg segmentation) according to standard taxonomic keys for Ixodid ticks [19,20,21,22]. Following identification, ticks were placed in individual sterile tubes and transported to the laboratory on ice; upon arrival, they were stored at −80 °C until further processing. All specimens were handled in accordance with biosafety guidelines for potentially infected arthropods to prevent contamination and ensure safety prior to the molecular analysis.

Ticks were washed with distilled water and mechanically homogenized in phosphate-buffered saline using glass beads (150–212 μm diameter) in a TissueLyser II cell disrupter (Qiagen, Hilden, Germany) at 40 Hz for 5 min to disrupt tissues and release intracellular content. Each tick was processed individually to prevent cross-contamination. Total RNA was extracted from 200 μL of supernatant of each tick homogenate using the QIAamp cador Pathogen Mini Kit (Qiagen, Hilden, Germany) following the manufacturer’s instructions. Negative extraction controls were included in each batch to monitor for potential contamination. Nucleic acids were eluted in 45 μL nuclease-free water and stored at −80 °C until further analysis.

Detection of CCHFV and AIGV RNA was performed using a commercial real-time RT-PCR kit (Congo Crimea Real-TM, Sacace Biotechnologies Srl, Como, Italy). Each run included a positive control, a negative template control, and an extraction control to validate assay performance. All positive tick samples were further tested by an in-house RT-nested PCR which amplifies a fragment of the small (S) RNA segment of the virus [23] Supplementary File S1. The PCR products were Sanger sequenced in a SeqStudio Genetic Analyser (Thermo Fisher Scientific Inc., Waltham, MA, USA). The nucleotide sequences were analyzed using the National Center for Biotechnology Information (NCBI) Basic Local Alignment Sequence Tool (BLAST) version 5 search engine (https://blast.ncbi.nlm.nih.gov/, accessed on 5 February 2025) to identify the best match.

Phylogenetic analysis was conducted to determine the genetic relationships between the sequences from this study and representative AIGV and CCHFV sequences retrieved from GenBank (NCBI). A maximum likelihood phylogenetic tree was constructed based on the best-fit nucleotide substitution model identified by model selection criteria. Branch support was assessed by bootstrap analysis with 1000 replicates. The resulting phylogenetic tree was visualized and annotated using MEGA 11 software [24].

Spatial visualization of the locations where *Hyalomma* spp. and *R. bursa* were collected was performed in RStudio (v2025.09.1). Sampling coordinates (in decimal degrees) from the dataset were mapped onto administrative boundaries at the RU level, sourced from the Hellenic Statistical Authority (ELSTAT) [25]. Separate maps were generated for *Hyalomma* spp. and *R. bursa* using distinct symbols; the locations with AIGV-positive ticks were indicated in red.

## 3. Results

### 3.1. Tick Collection

Of the 680 ticks collected in the study, 135 were identified as *Hyalomma* spp. (105 *H. excavatum*, 25 *H. marginatum*, and 5 *H. anatoliticum*), and 545 as *R. bursa.* All *Hyalomma* ticks were adults (55.6% male). Among the *R. bursa* ticks, 511 were adults (51.1% male) and 34 were nymphs. Table 1 and Table 2 summarize the distribution of *Hyalomma* spp. and *R. bursa* ticks, respectively, by Region, RU and source (host or environment).

### 3.2. Molecular Testing

Positive results from real-time RT-PCR were obtained exclusively from adult *R. bursa* ticks, while *R. bursa* nymphs and *Hyalomma* ticks tested negative. Specifically, 12 out of 511 (2.3%) adult *R. bursa* specimens tested positive, with Ct values ranging from 24.84 to 37.21. These samples were further tested by the in-house RT-nested PCR and resulted positive. Sequencing of the PCR products followed by BLAST analysis of the sequences showed that all positive ticks were carrying AIGV sequences.

AIGV-positive ticks were collected from two Regions: West Greece (at Aetolia-Acarnania RU) and Eastern Macedonia and Thrace (at Xanthi RU). Specifically, in West Greece, 7 out of 136 adult *R. bursa* ticks tested positive. These were collected in May (three from a goat and two from a sheep, on different farms in the same village), and June (one from a goat and one from a sheep from different farms and villages). In Eastern Macedonia and Thrace, 5 out of 58 *R. bursa* ticks tested positive; all 5 were collected in June from a single goat.

### 3.3. Phylogenetic Analysis

A 220 bp fragment sequence was taken from nine ticks (five from West Greece and four from Eastern Macedonia and Thrace). Within each Region, the sequences were identical. The two groups shared 99% nucleotide and 100% amino acid identity. Pairwise genetic distances at the nucleotide and amino acid levels between each group and the AIGV AP-92 and Pentalofos strains are shown in Table 3.

**Figure 1:**
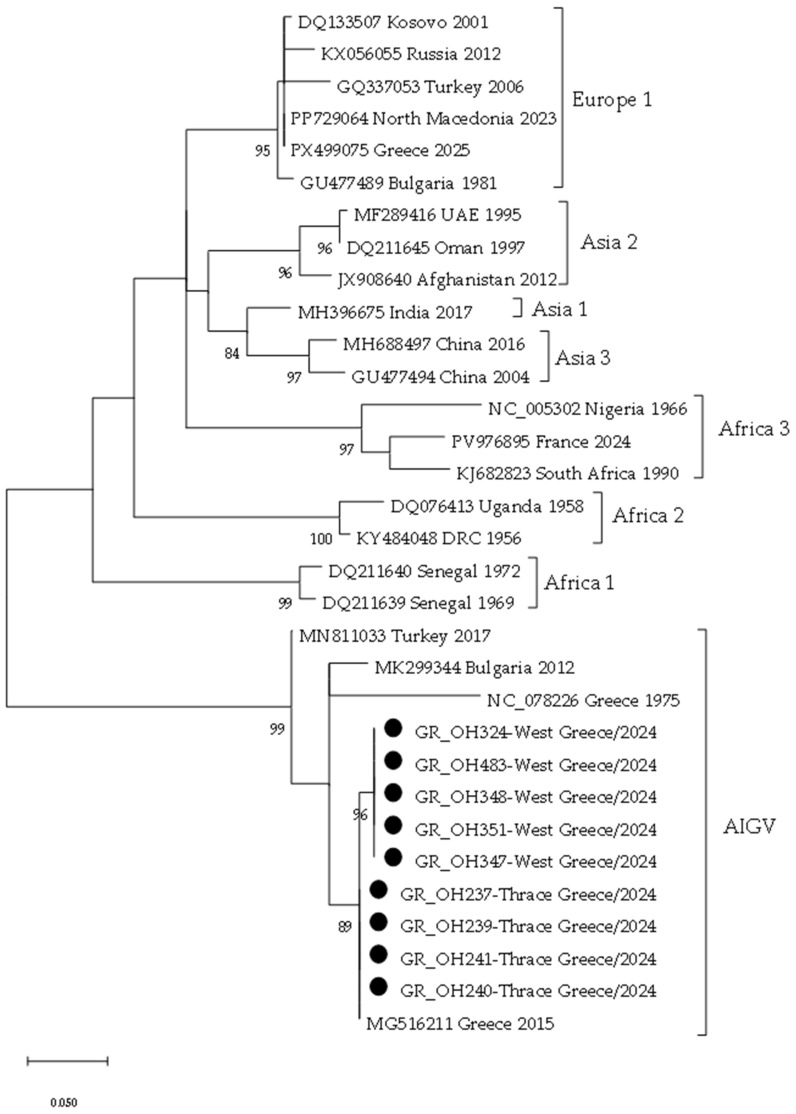
Maximum likelihood phylogenetic tree based on a 220 bp fragment of the AIGV and CCHFV S RNA segments. The tree was constructed using the Kimura 2-parameter model. Bootstrap values >80% are shown. Sequences are identified by accession number, country and year of detection. CCHFV genetic lineages are represented in the tree. AIGV sequences from this study are marked with black circles.

A maximum likelihood phylogenetic tree (Figure 1) was inferred from a 220 bp fragment sequence of the S RNA segment using the Kimura 2-parameter model. AIGV sequences from this study are marked.

### 3.4. Mapping

**Figure 2:**
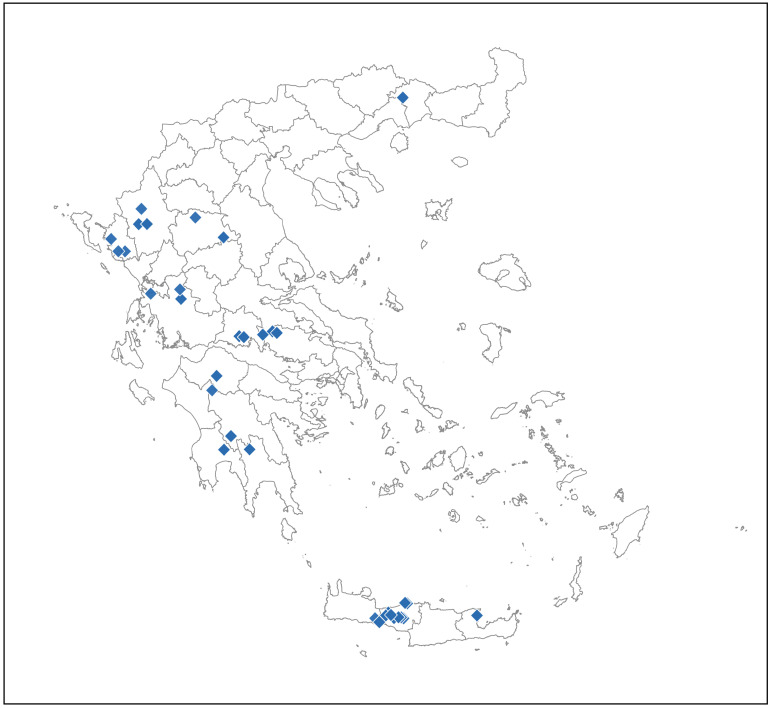
Map of Greece showing with blue squares the sampling locations of *Hyalomma* spp. ticks.

**Figure 3:**
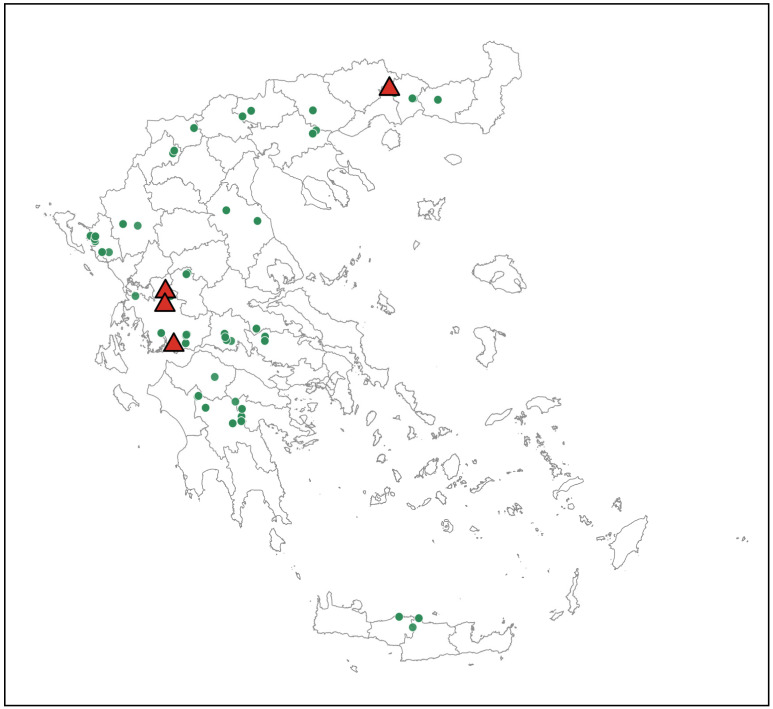
Map of Greece showing with green circles the sampling locations of *Rhipicephalus bursa* ticks. Red triangles indicate sites where AIGV-positive ticks were identified.

The geographic distribution of collected *Hyalomma* spp. and *R. bursa* ticks is illustrated in Figure 2 and Figure 3, respectively. Locations in the two RUs where AIGV-positive *R. bursa* ticks were detected are indicated by a red triangle. It should be noted that each location (e.g., village or town) may represent one or more screened farms.

## 4. Discussion

Molecular surveillance of vectors is essential for detecting vector-borne pathogens prior to the occurrence of clinical human cases, enabling early warning and outbreak preparedness [13,26]. Among tick-borne viral zoonoses in Europe, CCHF is of particular concern due to its severity and high case fatality rate [26]. France serves as an example of a European country with silent CCHFV circulation; the virus has been detected in ticks on the island of Corsica and in southern mainland France, despite the absence of human cases [27,28,29]. Consequently, screening ticks for CCHFV is essential for identifying hotspots of virus circulation, even in the absence of reported human cases, to increase preparedness and vigilance among healthcare and public health professionals.

In this study, CCHFV was not detected in the sampled ticks, while AIGV was detected in *R. bursa* ticks in two RUs in Greece. The overall detection rate of AIGV RNA in adult *R. bursa* ticks was 2.3%. In comparison, neighboring Albania reported AIGV in 13.3% of *R. bursa* and 1% of *H. marginatum* pools, while CCHFV (genotype Europe 1) was detected in 14.3% of *H. marginatum* pools, restricted to the endemic Kukes region [30]. In Kosovo, 9.3% of *R. bursa* ticks in a non-endemic area were AIGV-positive, while 11% of *H. marginatum* from hyper-endemic regions were CCHFV-positive [31]. Currently the exact pathogenicity of AIGV remains unknown. A few human mild cases have been documented in Türkiye, while a fatal case has been reported in Iran [32,33]. Future clinical studies will help to elucidate this field of research.

The observed temporal clustering of AIGV positive ticks in May and June aligns with the seasonal activity of adult *R. bursa* in Greece (May to September), reflecting the influence of temperature and humidity on tick population dynamics and virus transmission [34]. Similar seasonal patterns documented in other endemic countries highlight the importance of timing in tick surveillance strategies. The low *Hyalomma* count is likely due to host and ecological factors rather than geography. One possible explanation is that the ticks in our study were collected from sheep and goats, while *Hyalomma* adult ticks in the Mediterranean countries typically parasitise large ungulates [20,22]. Therefore, further surveillance involving large ungulates and wildlife may provide valuable data on the distribution of *H. marginatum* in Greece and the level of CCHFV circulation. However, it cannot be excluded that the current *H. marginatum* population in Greece is indeed low. It is noteworthy that prior to 2005 the proportion of *H. marginatum* ticks in Türkiye was less than 5%; however, in 2005, 74% of cattle were found to be infested with ticks, 85% of which were *H. marginatum* [35,36]. In 2002, the first CCHF cases were reported in Türkiye [37]; since then, CCHFV is causing annual outbreaks in the country.

CCHFV and AIGV belong to different species within the same genus (*Orthonairovirus*) [6]. Since the primers and probes used in molecular assays for CCHFV detection (both in-house and commercial) are generally based on conserved genomic regions, they often detect both viruses [38]. Consequently, sequencing is essential to achieve species-level resolution and accurate differentiation between these co-circulating orthonairoviruses. Cross-reactivity is also observed in serology, suggesting that exposure to AIGV may contribute to CCHFV positive results in serosurveys. Currently, specific serological assays capable of distinguishing between CCHFV and AIGV are not available.

The two viruses differ in their primary vectors, with CCHFV carried mainly by *Hyalomma* spp. ticks in Eurasia [36], whereas AIGV is most commonly associated with *Rhipicephalus* spp., mainly *R. bursa*. In this study, AIGV was detected in 2.3% of adult *R. bursa* ticks, while the *R. bursa* nymphs and *Hyalomma* ticks tested negative. As previously noted, surveillance studies focusing on ticks from large animals may provide additional data.

Twelve AIGV-positive ticks were detected at five sites in two RUs, one in the western and one in the northeastern part of Greece. The detection of AIGV-positive ticks on different farms in two geographically distant RUs highlights the focal and heterogeneous nature of virus circulation, as previously described for CCHFV [16].

In the phylogenetic tree, the detected AIGV sequences form a distinct subclade together with the AIGV Pentalofos strain (Acc. No MG516211) which was isolated in 2015 from *R. bursa* ticks in Greece (Figure 1). Since the tree is based on a short S RNA fragment, it lacks the resolution for deep evolutionary interpretation; thus, the clustering remains provisional. To accurately determine the genetic relationships of AIGV, a phylogenetic analysis based on whole-genome sequences of all three RNA segments is required.

AIGV has been detected in several CCHF endemic Balkan countries and Türkiye [30,31,39]. Specifically in Kosovo, tick surveillance revealed that species composition varies between endemic and non-endemic areas, with *H. marginatum* dominating in high CCHFV transmission zones, whereas *R. bursa* is more pevalent in mixed habitats [31]. These Balkan findings reinforce the view that vector composition and infection prevalence are driven by complex interactions among climate, land use, and host populations, shaping the local epidemiology of CCHFV and related orthonairoviruses.

## 5. Conclusions

AIGV was detected in adult *R. bursa* ticks in two RUs of Greece in 2024, while CCHFV was not detected in the sample set. The low number of collected *H. marginatum* ticks limits inference regarding CCHFV circulation. The recent report of human CCHF cases in Greece suggests that co-circulation of CCHFV and AIGV cannot be ruled out. Therefore, One Health surveillance of ticks, humans, and animals (domestic and wild) is essential to better understand the complex epidemiology of CCHF in Greece. Furthermore, clinical research is needed to elucidate the pathogenicity and virulence of AIGV and its broader impact on public health.

## Figures and Tables

**Table 1:** Number of *Hyalomma* spp. ticks collected by Region, regional unit, and source (host).

Region	Regional Unit	Source			Number of Ticks
		Goats	Sheep	Cattle	
East Macedonia and Thrace	Xanthi	0	2	0	2
Epirus	Thesprotia	0	3	0	3
	Ioannina	0	9	0	9
Thessaly	Trikala	0	1	2	3
Central Greece	Boeotia	3	7	0	10
	Phocis	0	6	0	6
West Greece	Aetolia-Acarnania	0	3	1	4
	Achaea	0	2	0	2
Peloponnese	Arcadia	0	5	0	5
	Laconia	0	4	0	4
	Messenia	0	24	0	24
Crete	Rethymno	10	38	0	48
	Lasithi	0	15	0	15
Total		13	119	3	135

**Table 2:** Number of *Rhipicephalus bursa* ticks collected by region, regional unit, and source (host or environment).

Region	Regional Unit	Source				Number of Ticks
		Goats	Sheep	Cattle	Environment	
East Macedonia and Thrace	Xanthi	52	6	0	0	58
	Rhodope	5	0	0	0	5
Central Macedonia	Thessaloniki	5	0	0	0	5
	Kilkis	14	7	0	0	21
	Serres	0	3	0	0	3
West Macedonia	Kastoria	9	6	0	0	15
	Florina	31	14	0	0	45
Epirus	Thesprotia	0	26	0	0	26
	Ioannina	1	7	0	0	8
Thessaly	Trikala	17	0	0	0	17
	Larissa	7	0	0	0	7
Central Greece	Boeotia	9	0	0	1	10
	Phthiotis	14	0	0	0	14
	Evrytania	0	7	0	0	7
	Phocis	0	8	0	2	10
West Greece	Aetolia- Acarnania	66	72	1	0	139
	Achaea	0	1	0	0	1
Peloponnese	Argolis	0	6/5	0	0	6
	Arcadia	84	44	0	9	137
Crete	Rethymno	2	1	0	0	3
	Heraklion	0	8	0	0	8
Total		315	216	1	12	545

**Table 3:** Pairwise genetic distances (%) at the nucleotide (nt) and amino acid (aa) levels between the two AIGV groups of the study and the AIGV strains AP-92 and Pentalofos.

AIGV Group	AP-92 Strain (NC_078226)		Pentalofos Strain (MG516211)	
	nt	aa	nt	aa
West Greece	10.33	1.4	0.92	0.00
Eastern Macedonia and Thrace	10.37	1.4	0.00	0.00

## Data Availability

The sequences of this study have been deposited in the GenBank DataBase and received the accession numbers PZ190057-PZ190064.

## Supplementary Materials

The following supporting information can be downloaded at: https://www.mdpi.com/article/10.3390/v18050483/s1, File S1: Method.
