# Supplementary material for: Screening Ticks for Crimean–Congo Hemorrhagic Fever Virus and Aigai Virus in Greece
Source: Viruses. 2026 Apr 22;18(5):483. doi: 10.3390/v18050483 (PMC13211716; doi:10.3390/v18050483)
Supplement: Supplementary file 1 [file viruses-18-00483-s001.zip › viruses-4219264-supplementary.pdf]

Method

RT-nested PCR

All positive ticks were tested by an in-house RT-nested PCR which amplifies a fragment of the small (S) RNA segment of Crimean-Congo hemorrhagic fever virus [23].

The RT- PCR was conducted in a 50-μl reaction mixture containing 1X buffer, 1.8 mM $MgCl_2$, 0.8 μM of deoxynucleoside triphosphates (dNTPs), 3.5 μM primer F2 (5′-TGGACACCTTCACAAACTC-3′), 3.5 μM primer R3 (5′-GACAAATTCCCTGCACCA-3′), 13 U reverse transcriptase, 3 U recombinant Taq DNA polymerase (Invitrogen, Thermo Fisher Scientific, MA, USA) and 5 μl of the extracted RNA. The cycling conditions were 41°C for 60 min for the reverse transcription; 95°C for 2 min for denaturation; 40 cycles of 94°C for 30 s, 47°C for 1 min, and 72° for 2 min for amplification; a final extension at 72° for 5 min.

The solution mix of the nested PCR consisted of 1X buffer; 1.8 mM $MgCl_2$, 0.8 μM of dNTPs, 3.5 μM of primer F3 (5′-GAATGTGCATGGGTTAGCTC-3′), 3.5 μM of primer R2 (5′-GACATCACAATTTCACCAGG-3′), 2.5 U DNA polymerase and 2 μl from the first round PCR product. The cycling conditions were: 95°C for 2 min; 40 cycles of 95°C for 30 s, 41°C for 1 min, and 72° for 2 min; and 72° for 5 min. The PCR products were visualized by ethidium bromide staining after 1.5% agarose gel electrophoresis. The expected size of the PCR products was 535 bp from the first round, and 260 bp from the second round PCR.
